# Discussing study limitations in reports of biomedical studies- the need for more transparency

**DOI:** 10.1186/1477-7525-10-23

**Published:** 2012-02-23

**Authors:** Milo A Puhan, Elie A Akl, Dianne Bryant, Feng Xie, Giovanni Apolone, Gerben ter Riet

**Affiliations:** 1 Department of Epidemiology, Johns Hopkins Bloomberg School of Public Health, 615 North Wolfe Street, Mail room E6153, Baltimore, MD 21205, USA; 2 Department of Medicine, State University of New York at Buffalo, Buffalo, NY, USA; 3 Faculty of Health Sciences, University of Western Ontario, London, Canada; 4 Department of Clinical Epidemiology and Biostatistics, McMaster University, Hamilton, Canada; 5 Scientific Directorate, Arcispedale Santa Maria Nuova, IRCCS, Reggio Emilia, Italy; 6 Department of General Practice, Academic Medical Center University of Amsterdam, Amsterdam, The Netherlands

## Abstract

Unbiased and frank discussion of study limitations by authors represents a crucial part of the scientific discourse and progress. In today's culture of publishing many authors or scientific teams probably balance 'utter honesty' when discussing limitations of their research with the risk of being unable to publish their work. Currently, too few papers in the medical literature frankly discuss how limitations could have affected the study findings and interpretations. The goals of this commentary are to review how limitations are currently acknowledged in the medical literature, to discuss the implications of limitations in biomedical studies, and to make suggestions as to how to openly discuss limitations for scientists submitting their papers to journals. This commentary was developed through discussion and logical arguments by the authors who are doing research in the area of hedging (use of language to express uncertainty) and who have extensive experience as authors and editors of biomedical papers. We strongly encourage authors to report on all potentially important limitations that may have affected the quality and interpretation of the evidence being presented. This will not only benefit science but also offers incentives for authors: If not all important limitations are acknowledged readers and reviewers of scientific articles may perceive that the authors were unaware of them. Authors should take advantage of their content knowledge and familiarity with the study to prevent misinterpretations of the limitations by reviewers and readers. Articles discussing limitations help shape the future research agenda and are likely to be cited because they have informed the design and conduct of future studies. Instead of perceiving acknowledgment of limitations negatively, authors, reviewers and editors should recognize the potential of a frank and unbiased discussion of study limitations that should not jeopardize acceptance of manuscripts.

## Introduction

The physicist Richard Feynman argued, during his commencement address at the California Institute of Technology in 1974, that utter honesty must be a cornerstone of scientific integrity. He cautioned researchers from fooling themselves by saying: "We've learned from experience that the truth will come out. Other experimenters will repeat your experiment and find out whether you were wrong or right. Nature's phenomena will agree or they'll disagree with your theory. And, although you may gain some temporary fame and excitement, you will not gain a good reputation as a scientist if you haven't tried to be very careful in this kind of work."[1]

We think that, in today's culture of publishing biomedical studies, many authors may not want to discuss limitations of their studies because they perceive a transparency threshold beyond which the probability of manuscript acceptance goes down (perhaps even to zero) [2]. The goals of this commentary are to briefly review how limitations are currently acknowledged in the biomedical literature, to discuss implications of limitations in biomedical studies, and to make suggestions as to how to openly discuss limitations for scientists who submitting their papers to biomedical journals. This commentary was initiated by two of the authors (MP and GtR), who are doing research in the area of hedging (use of language to express uncertainty), and proposed to the editors of *Health and Quality of Life Outcomes*. All editors supported the idea of writing a commentary on the importance of discussing limitations transparently and four editors (EAA, DB, FX, GA) joined the writing group. This commentary was developed through discussion and logical arguments by the authors who have extensive experience as authors and editors of biomedical papers themselves.

Recognition, acknowledgment and discussion of all potentially important limitations by authors, if presented in an unbiased way, represent a crucial part of the scientific discourse and progress. The advantages of openly discussing limitations are probably long-term and benefit the scientific community and other users of the evidence: A candid discussion of limitations helps readers to correctly interpret the particular study. Conflicting results across studies may be explained by the patterns in limitations. Moreover, frank discussion of limitations informs future studies, which are likely to be of higher quality if they address the limitations of earlier studies. However, while encouraging others to openly discuss limitations of their studies is easy, discussing the limitations of one's own study is more challenging. Researchers usually have their opinion about how to design and execute studies or how to interpret the results and may not agree that some aspects of a study represent, in the view of others, a limitation. Risks of acknowledging limitations and having an open scientific discourse may include, at least in today's culture, eliciting negative comments by peer reviewers, non-acceptance by journals and a potentially negative image as a researcher.

### Discussion of limitations in the medical literature

There is some evidence that limitations are not thoroughly discussed in the medical literature. A study using automated key word searching found that only 17% out of 400 papers published in leading medical journals used at least one word referring to limitations [3]. Not a single article discussed how a limitation could affect the conclusion. In a more detailed assessment of the medical literature, in which two independent reviewers assessed the abstract and discussion sections of 300 medical research papers, published in first and second tier general medical and specialty journals, 73% of all papers were found to acknowledge a median of 3 limitations [4]. This higher proportion (compared to the first study) is likely due to a more thorough assessment (i.e., by reviewers rather than an automated search) but could also be related to a slightly different selection of papers. The detailed assessment of these 300 papers revealed that 62% of all limitations referred to aspects of internal validity, which could systematically distort the results. Measurement errors, failure to measure important variables and potential confounding were among those acknowledged most frequently. The remaining limitations referred to aspects of applicability of the results to clinical practice (external validity). Differences between the study population and real-world populations were mentioned most frequently as barriers for applying the results in practice. Few authors discussed how the limitations could have affected the interpretation of study findings.

What is currently unclear is whether authors do or do not address those limitations that are most likely to affect internal validity and applicability of results in real practice. It may well be possible that authors discuss limitations because it is required by journal policies and worry that too open discussion jeopardizes the chances of acceptance. Also, more research is needed to see how the acknowledgment of potentially important limitations fits with the claims made in an article, for example about the effectiveness of a medical intervention or about the measurement properties of a patient-reported outcome.

It is time to discuss limitations not in isolation but in the context of the entire article and as part of a rhetorical-epistemic phenomenon that linguists call "hedging." Hedging refers to "the means by which writers can present a proposition as an opinion rather than a fact" [5]. By using hedging authors can express the extent of uncertainty about the importance and validity of their study but also prevent readers from making false accusations for strong or definitive statements. Of note, hedging has both positive and negative connotations since it can be used to set an appropriate tone but also to express an opinion that may not be fully supported by the facts.

### Discussing implications of limitations prevents misunderstandings and supports interpretation of data

It requires a great deal of judgment to estimate the potential impact of limitations on internal or external validity of a study. Sometimes, the direction of bias may be towards an over- or underestimation of effects. For example, if there is systematic measurement error that equally affects different study groups (so called non-differential measurement error, for example if the exposure is measured with a sensitivity of 80% and a specificity of 90%) the results are usually biased towards an underestimation of the effect [6]. Or, if a confounder is positively associated with the outcome and more prevalent in study participants exposed to the risk factor of interest, an overestimation of the effect can be expected. Some biases, for example selection bias and some forms of measurement error can, affect the results in a direction that is difficult to predict [6]. Sometimes, the impact of biases on internal validity may be so small that its description may not be warranted.

Very often the authors of an article are in the best position to judge the direction of a potential bias because they executed the study and have experienced first-hand limitations of their study. In addition, they often have the needed content knowledge that would inform the direction and potential extent of bias. Thus authors should acknowledge recognized limitations and discuss their likely implications on the interpretations of the findings; by doing so, they reduce the probability that readers will misjudge the validity and impact of their study. Of course, it is important that the authors also include the reasoning behind their judgment of the magnitude and direction of the potential bias to enable readers to form their own opinion on the impact of limitations.

For some limitations, however, the impact can better be judged in a meta-epidemiological context, that is, when all studies addressing the same research questions are analyzed together. Some journals ask authors to discuss their results in reference to an existing systematic review [7]. Thereby, not only heterogeneity of results across studies can be detected but it may be possible to estimate how much a limitation may affect the results. For example, a randomized trial may use a generic health-related quality of life instrument to evaluate the effectiveness of a treatment. The trial may show no effect and have high internal validity. However, other trials evaluating the same treatment may have used a disease-specific instrument and shown an effect that exceeded the minimal important difference. Or, studies may have shown that disease-specific instruments discriminate better between disease severity or change over time than generic instruments [8,9]. The limitation of the first trial that used a much less responsive generic instrument only becomes much clearer in a meta-epidemiological context. Another important purpose of systematic reviews is to identify limitations of existing studies and to help investigators to avoid them in the future. It is beyond the scope of this commentary to discuss different types of biases and their implications for the quality of evidence but we refer readers to the extensive literature on biases and to some approaches that are currently used to judge the implications of limitations on the strength of evidence [6,10-12].

### An open discussion of limitations should not jeopardize paper acceptance by journals

We would like to strongly encourage authors submitting their articles to biomedical journals to openly discuss all potentially relevant limitations of their study. Specifically, we suggest including text in the abstract and discussion section (Table 1):

**Table 1:** Suggestions for discussing limitations of studies more transparently

Section of paper	Suggestions
**Abstract**	At the end of the results section add one sentence highlighting the one or two main limitations of the study
**Discussion**	Report on all limitations that may have affected the quality of the evidence being presented, including aspects of study design and implementation.
	Give the authors' view on how the limitations impact on the quality of the evidence and discuss the direction and magnitude of bias
	Do not restrict the discussion of limitations to aspects of internal validity and discuss where the limits of applicability of the results may lie
	Discuss the strengths of the study that may counterbalance or outweigh (some of) the limitations.
	Provide suggestions for future research specifically overcoming the limitations of the current study.

## Abstract

At the end of the results section add one sentence highlighting the one or two main limitations of the study. The conclusion section should reflect the seriousness of the limitations as perceived by the authors and their potential impact on the results and interpretation of the study.

## Discussion section

1. Report on all limitations that may have affected the quality of the evidence being presented, including aspects of study design and implementation. Readers depend on a candid communication by the authors and may get the impression that the investigators were naive if they are not reported. If space is limited an online appendix could be considered that describes the limitations as well as their potential implications in more details.

2. Give the authors' view on how the limitations impact on the quality of the evidence and discuss the direction and magnitude of bias. For example, a recent study reporting on the association of quality of life of elderly people with nursing home placement and death discussed the potential mechanism of a selection bias by economic status. The authors concluded that a selection bias based on economic status was unlikely because access to health care, and thus selection into the study, did not depend on economic status [13]. As explained above, few authors currently discuss how limitations could have affected the strength of the conclusions that may be drawn. However, the authors should take advantage of their content knowledge and familiarity with the study and the meta-epidemiological context to prevent misinterpretations of the limitations by reviewers and readers.

3. Do not restrict the discussion of limitations to aspects of internal validity. For readers, it is important to learn about potential barriers for applying the evidence, generated in scientific studies, to practice. Discuss where the limits of applicability of the results may lie. This requires a discussion of the setting in which the study took place, how and why the results may differ in another setting (potential effect modification) and what barriers may exist to adopt new interventions or diagnostic procedures in a setting that is different from the research setting [14].

4. Discuss the strengths of the study that may counterbalance or outweigh (some of) the limitations. Be explicit about the strengths, in particular how the study was implemented, and do not limit the discussion of strengths to general statements about study design.

5. Provide suggestions for future research specifically overcoming the limitations of the current study. One may also consider describing how one's own study could be repeated and conducted differently to avoid some of the limitations. Articles acknowledging and putting into context all potentially relevant limitations could help shape the research agenda and may be more likely to be cited because they inform the design and conduct of future studies.

We acknowledge that, even if limitations are openly discussed, some articles will be rejected by journals because the limitations affect an article's validity, level of interest to the reader and comprehensibility too much as assessed by peer reviewers. But we believe that journal editors should consider the thoroughness with which limitations are discussed in their editorial decisions on acceptance. In fact, editors should consider it a shortcoming of the submission if a candid discussion is lacking. To end with Feynman's words, "[...] if you are doing an experiment, you should report everything that you think might make it invalid - not only what you think is right about it: other causes that could possibly explain your results; and things you thought of that you've eliminated by some other experiment, and how they worked - to make sure the other fellow can tell they have been eliminated."[1]
